# Developmental atlas of the RNA editome in *Sus scrofa* skeletal muscle

**DOI:** 10.1093/dnares/dsz006

**Published:** 2019-04-23

**Authors:** Yalan Yang, Min Zhu, Xinhao Fan, Yilong Yao, Junyu Yan, Yijie Tang, Siyuan Liu, Kui Li, Zhonglin Tang

**Affiliations:** 1 Research Center for Animal Nutriomics at Shenzhen, State Key Laboratory of Animal Nutrition, Agricultural Genomics Institute at Shenzhen, Chinese Academy of Agricultural Sciences, Shenzhen, China; 2 Genome Analysis Laboratory of the Ministry of Agriculture, Agricultural Genomics Institute at Shenzhen, Chinese Academy of Agricultural Sciences, Shenzhen, China; 3 Institute of Animal Sciences, Chinese Academy of Agricultural Sciences, Beijing, China; 4 Team of Pig Genome Design and Breeding, Research Centre of Animal Genome, Agricultural Genomics Institute at Shenzhen, Chinese Academy of Agricultural Sciences, Shenzhen, China

**Keywords:** RNA editing, skeletal muscle, development, pig, transcriptome

## Abstract

Adenosine-to-inosine (A-to-I) RNA editing meditated by adenosine deaminases acting on RNA (ADARs) enzymes is a widespread post-transcriptional event in mammals. However, A-to-I editing in skeletal muscle remains poorly understood. By integrating strand-specific RNA-seq, whole genome bisulphite sequencing, and genome sequencing data, we comprehensively profiled the A-to-I editome in developing skeletal muscles across 27 prenatal and postnatal stages in pig, an important farm animal and biomedical model. We detected 198,892 A-to-I editing sites and found that they occurred more frequently at prenatal stages and showed low conservation among pig, human, and mouse. Both the editing level and frequency decreased during development and were positively correlated with ADAR enzymes expression. The hyper-edited genes were functionally related to the cell cycle and cell division. A co-editing module associated with myogenesis was identified. The developmentally differential editing sites were functionally enriched in genes associated with muscle development, their editing levels were highly correlated with expression of their host mRNAs, and they potentially influenced the gain/loss of miRNA binding sites. Finally, we developed a database to visualize the *Sus scrofa* RNA editome. Our study presents the first profile of the dynamic A-to-I editome in developing animal skeletal muscle and provides evidences that RNA editing is a vital regulator of myogenesis.

## 1. Introduction

RNA editing represents a widespread post-transcriptional modification that enhances transcriptome diversity by changing RNA sequences.^1^ The extensive application of next-generation sequencing has greatly facilitated the identification of RNA editing. An astonishingly large number of RNA editing sites have been confidently detected in humans, mice, and other species, demonstrating that RNA editing is pervasive in the genome.^2–7^ Adenosine-to-inosine (A-to-I) RNA editing is the most prevalent form of RNA editing in mammals. This process is catalysed by the adenosine deaminases acting on RNA family of enzymes (ADARs).^8^ Following deamination of adenosine to inosine (I) by ADARs, the modified base is recognized as guanosine (G) by the cellular machinery during translation.^1^^,^^9^ It is probable that A-to-I RNA editing in protein-coding regions may result in amino acid sequence changes that affect other transcriptional processes in particular tissues or developmental stages.^1^^,^^9^ Amino acid changes caused by A-to-I editing may influence the propagation of fast electrical and chemical signals transmitted via ligand- and voltage-gated ion channels or neurotransmitter receptors in animal nervous systems.^10^^,^^11^ In addition, A-to-I RNA editing is essential for normal vertebrate development because it regulates gene expression by affecting alternative splicing, microRNA target recognition, and other biological processes.^12–16^

The pig (*Sus scrofa*) is an important protein source for humans and a widely used model organism in biomedical research.^9^^,^^17–19^ In comparison with rodents, pigs have a longer gestational period (about 114 days). Since it is difficult to collect prenatal skeletal muscle samples from humans and rodents, the pig is a valuable model organism for studying skeletal muscle development.^20–22^ Moreover, pigs share anatomical, developmental, physiological, metabolic, and genomic properties with humans.^17^ Skeletal muscle development is a highly complex and genetically well-programmed process controlled by cascades of myogenesis genes.^23^^,^^24^ Recent studies suggest that RNA editing contributes to myogenesis^25^ and that the editing level in skeletal muscle is relatively low compared with that of other tissues.^2^^,^^26^ ADAR enzymes catalyse the deamination of adenosine to inosine and play important roles in the myoblast-to-myotube transition because they are targets of muscle-specific myomiRs such as miRNA-1/206.^25^ AIMP2 inhibits RNA editing in muscle cells by promoting degradation of ADAR1/2 proteins.^2^ These observations imply that A-to-I editing plays an essential role in myogenesis. Currently, the mammalian skeletal muscle editome and the manner in which it changes during development are poorly understood.^26^^,^^27^

Here, we constructed a comprehensive dynamic atlas of the *S. scrofa* RNA editome in skeletal muscle across 27 developmental stages by integrating whole genome sequencing (WGS), genome-wide bisulphite sequencing (WGBS), and strand-specific rRNA-depleted total RNA sequencing (RNA-seq) data. To our knowledge, this is the first systematic study of RNA editing in skeletal muscle. The findings of this study suggest that RNA editing is a vital, but underappreciated, mechanism involved in regulating skeletal muscle development.

## 2. Materials and methods

### 2.1 Sample collection

Skeletal muscle (*longissimus dorsi*) samples were collected from Landrace (L) pigs at 27 developmental stages, including embryonic days 33, 40, 45, 50, 55, 60, 65, 70, 75, 80, 85, 90, 95, 100, and 105 (abbreviated as LE33, LE40, LE45, LE50, LE55, LE60, LE65, LE70, LE75, LE80, LE85, LE90, LE95, LE100, and LE105) and postnatal days 0, 9, 20, 30, 40, 60, 80, 100, 120, 140, 160, and 180 (abbreviated as LD0, LD9, LD20, LD30, LD40, LD60, LD80, LD100, LD120, LD140, LD160, and LD180). The experimental pigs were allowed access to feed and water *ad libitum* and were housed under identical conditions before slaughtering. After copulation with the boar, the sows and piglets were sacrificed at a commercial slaughter house at the selected stages. At each stage, skeletal muscle samples from three pigs were harvested as biological replicates. All samples were stored immediately in liquid nitrogen. All animal procedures were performed according to the protocols of the Chinese Academy of Agricultural Sciences and the Institutional Animal Care and Use Committee.

### 2.2 Construction of RNA-seq, WGS, and WGBS libraries

For the RNA-seq experiments, total RNA was isolated using TRIzol Reagent (Invitrogen), followed by rRNA depletion and DNaseI treatment (Qiagen). Strand-specific RNA-seq libraries for paired-end sequencing were prepared using the Illumina Ribo-Zero protocol. For each stage, three independent biological libraries were constructed. The libraries were sequenced to generate paired-end reads with 150-bp read lengths using the Illumina HiSeq X Ten platform. Approximately 100 million reads were acquired for each sample.

For WGS, genomic DNA was isolated from the ear tissue of a Landrace male pig with the same genetic background as the animals from which the samples used for RNA-seq was collected. The DNeasy Blood & Tissue Kit (Qiagen) was used for isolation of DNA according to the manufacturer’s instructions. Qualified DNA was sonicated into 350-bp fragments using a Covaris S220 ultrasonicator (Covaris). Libraries were constructed according to the standard operating procedure provided by Illumina. Sequencing was performed on an Illumina HiSeq 4000 platform, and 125-bp paired-end reads were generated.

The samples from the RNA-seq experiment were also used for WGBS. After DNA extraction, DNA was fragmented into 200–300 bp fragments with a Covaris S220 ultrasonicator (Covaris), followed by end repair and A-ligation. Cytosine-methylated barcodes were ligated to the sonicated DNA according to the manufacturer’s instructions. The DNA fragments were treated twice with bisulphite using the EZ DNA Methylation-Gold™ Kit (Zymo Research). The libraries were prepared according to the manufacturer’s instructions (Illumina). The libraries were sequenced on an Illumina HiSeq X Ten platform, and 150-bp paired-end reads were generated by Novogene (Novogene, Beijing, China).

### 2.3 Sequencing mapping and single-nucleotide variation calling

For each library, high-quality reads were obtained after removing reads containing adapters, reads containing poly-N sequences, and low-quality reads using in-house Perl scripts. The remaining clean, high-quality reads were used for subsequent analyses.

The RNA and DNA reads were aligned to the *S. scrofa* reference genome (downloaded from Ensembl, v11.1) by BWA (v0.7.17). The paired reads were mapped separately using the commands ‘bwa aln’ and ‘bwa sampe’, with only four mismatches allowed. Reads that uniquely mapped (*q* > 10) to the reference genome were kept by Samtools (v1.6).^28^ PCR duplicates that mapped to the same locations were removed by the MarkDuplicates tool from the Picard software package (v2.17.0), and only the read with the highest mapping quality was retained. The unique reads were subjected to local realignment around indels and base quality score recalibration using the IndelRealigner and BaseRecalibrator tools, respectively, from the Genome Analysis Toolkit (GATK, v3.4). The HaplotypeCaller tool from GATK was used to call variants.

For WGBS reads, the *S.* *scrofa* reference genome was first transformed to a bisulphite-converted version (C-to-T and G-to-A conversion) and then indexed using bowtie2.^29^ Bismark software (v 0.12.5) was used to perform alignments of bisulphite-treated reads to the reference genome using the default parameters.^30^ The sequencing depth and coverage of methylcytosine were summarized after removing duplicate reads. Bis-SNP software was used to call variants for the WGBS data.^31^

Reliable SNP sites were identified using stringent criteria, which required at least 10 sequencing reads at these sites and a high-quality score (*q* > 25). Sites with two or more variants were discarded. At least three reads with high quality were required to support the variant form to eliminate false positives due to amplification bias or sequencing error.

### 2.4 Identifying RNA editing sites

In order to accurately identify RNA editing sites in pig skeletal muscle, a computational pipeline was developed to make reliable calls on RNA editing sites (Supplementary Fig. S1) as reported in previous studies,^2^^,^^32–34^ but with slight modifications. In brief, to remove false-positive RNA editing sites, the variants called by RNA-seq were filtered by the following steps: (i) SNPs that were genotyped as heterozygous variants using the WGS and WGBS data were discarded; (ii) All known SNPs present in the SNP database were filtered out (dbSNP; database version 150, ftp://ftp.ensembl.org/pub/release-91/variation/vcf/sus_scrofa/); (iii) Intronic sites that occurred within 4 bp of splice junctions were discarded; (iv) Variants in homopolymer runs were discarded; (v) Because of the relatively high error rate of Illumina sequencing towards both ends of a read, variants that occurred within 6 bp of both ends of a read were removed; (vi) The remaining candidate sites were checked with BLAT alignment filtering, and sites in regions highly similar to other regions of the genome were discarded. The remaining variants were considered as candidate RNA editing sites and annotated by snpEff (v4.3t)^35^ based on Ensembl gene annotation (release 90).

### 2.5 Data analysis

For an RNA editing site in a given skeletal muscle sample, the A-I RNA editing level was quantified as the ratio of the number of G reads to the total number of A and G reads covering the site. The overall editing rate of each sample was determined as the total number of reads with G at all candidate editing positions compared with all A and G reads covering the editing positions.^2^ For the multidimensional scaling (MDS) analysis, we removed all sites that were missing editing measurements in more than 27 (one-third) of the samples. Only editing sites in transcript regions were used for the MDS analysis. The missing values of the editing sites were imputed using the missForest R package with default settings. The ‘*cmdscale*’ function in R was used for the MDS analysis.

A cluster of editing sites was defined as the occurrence of three or more candidate sites within a 100-bp window. Genes that contained at least five editing sites and one clustered region were defined as hyper-edited genes. The sequence spanning 200 bp upstream and downstream of the editing site or cluster was used to predict the secondary structure and to calculate the minimum free energy using the RNAfold programme in the ViennaRNA package.^36^ The binding sites and energies of miRNAs were predicted using Miranda software (v3.3a)^37^ with default values. Mature miRNA sequences of *S. scrofa* were obtained from miRBase (release 21).^38^ Two types of sequences with regions flanking (50 bp upstream and downstream) the editing sites were prepared for target prediction: reference sequences and A-to-I editing sequences.

For gene expression analysis, RNA-seq reads were aligned to the reference genome using Hisat2 (v2.0.4) with the parameter –rna-strandness RF, while the other parameters were set to their default values. The expression levels of genes were quantified by calculating fragments per kilobase of exon per million fragments (FPKM) values using StringTie (v1.3.1) in a reference-based approach.^39^ Gene annotation file was obtained from Ensembl (release 90). Gene ontology (GO) analysis was performed using the Database for Annotation, Visualization, and Integrated Discovery (DAVID) website (v6.7, http://david.abcc.ncifcrf.gov/).^40^

For conservation analysis, we converted the coordinates of all pig A-to-G RNA editing sites to positions on the human and mouse reference genomes using liftOver tools and the chain files (susScr11ToHg38.over.chain.gz and susScr11ToMm10.over.chain .gz) provided by the UCSC Genome Browser (https://genome.ucsc.edu). The known human and mouse A-to-I editing sites were downloaded from the RADAR (v2, http://rnaedit.com/)^41^ and REDIportal (http://srv00.recas.ba.infn.it/atlas/)^42^ databases. The human A-to-I editing sites in these two databases were merged. In total, 4,627,557 human A-to-I editing sites and 8,824 mouse A-to-I editing sites were used for the conservation analysis.

### 2.6 Editing changes and co-editing network analysis

A-to-I editing sites with less than one-third of values missing were selected from the 81 samples and used to identify differentially editing sites to ensure adequate statistical power. A multiple linear regression model with development stage and sex was developed to identify editing changes associated with skeletal muscle development or sex. Edited sites were considered to be significantly associated with skeletal muscle development or sex if they passed the FDR-corrected significance threshold of *P *≤* *0.05.

Weighted gene co-edited network analysis (WGCNA)^43^ was used to identify distinct modules of co-edited sites. A total of 9,478 editing sites with no missing values and editing in more than 10 samples (editing level >0) were used to construct co-editing modules. We chose the best soft-thresholding 4, which was the lowest power for which the scale-free topology fit index reached the *R*^2^ cut-off (>0.9). Modules with eigengenes that were highly correlated (above 0.8) were merged to assess their relationship with skeletal muscle development. We identified 13 co-editing modules with minModuleSize = 30. The correlations between module eigengenes and phenotypic traits (development stage and sex) were used to identify modules associated with development or sex.

### 2.7 Database construction

The database was composed of a web interface and a MariaDB database engine, which is used to store and manage all data. The web interface is composed of native HTML elements. The data processing and customer/service interaction programmes were written in PHP (v5.4.16) and JavaScript (v1.10.2). The search results are shown by asynchronous JavaScript and XML (AJAX) serves belonging to JavaScript. Baidu ECharts (http://echarts.baidu.com/index.html) are used to exhibit the editing level of editing sites at 27 developmental stages of skeletal muscle. The web services were built using nginx (v2.10.0), an HTTP and reverse proxy server. JBrowse (version 1.14.2) genome browser is used to display the positional relationships between genes and genome annotations.^44^

### 2.8 Validation of RNA editing by Sanger sequencing

To validate the reliability of RNA editing events identified by RNA-seq and bioinformatics analysis, we randomly selected 18 regions containing 104 editing sites for PCR validation. The gDNA and RNA samples used for PCR validation were those used for RNA-seq. Total RNA was firstly reverse-transcribed into cDNA using the RevertAid First Strand cDNA Synthesis Kit (Thermo, Waltham, MA, USA) according to the manufacturer’s protocols. The selected regions were amplified from the gDNA and cDNA samples using the primers listed in Supplementary Table S1. The PCR products were then subjected to Sanger sequencing. To verify the RNA editing level calculated from the RNA-seq reads, the PCR products were gel-purified using a Gel Extraction Kit (Tiangen, Beijing, China) and subcloned into the pMD18-T vector (Takara, Osaka, Japan) to produce TA clones. Finally, 42–50 clones from each sample were randomly picked for Sanger sequencing. The editing level of each editing site was calculated as the number of clones with the edited nucleotides compared with the total number of sequenced clones.

### 2.9 Western blot analysis

Total protein was isolated from pig skeletal muscle using a Protein Extraction Kit (TransGen Biotech, China). Western blotting was performed as previously described.^45^ The anti-ADAR1 antibody (SC-73208) and anti-ADAR2 antibody (Abp50601) were obtained from Cell Signaling Technology (Danvers, MA, USA) and Amy Jet Scientific (Guangzhou, China), respectively. The anti-GAPDH antibody (bs-2188R) and secondary antibody (bs0295G-HRP) were obtained from Bioss (Beijing, China). Densitometric analysis of bands was performed using ImageJ software (National Institutes of Health, Bethesda, MD, USA).

### 2.10 Vector construction, cell culture, and dual luciferase reporter assay

Three editing sites in the 3′-UTRs of the *AVPR1A*, *TMX4*, and *ACTN2* genes (chr5:27,827,411, chr17:16,833,004, and chr14:54,710,988) were randomly selected to verify whether A-to-I editing can affect miRNA binding. The 3′-UTR fragments flanking the miRNA binding sites of these three genes (without editing, 3′-UTR-wt) were amplified by PCR. The PCR products were cloned into the pmirGLO Dual-Luciferase Vector using the *Sac*I and *Xho*I restriction sites. The mutant types corresponding to the RNA editing sites (3′-UTR-edt) in these three genes were made by site-directed mutagenesis (Takara) and confirmed by Sanger sequencing. The primer sequences are listed in Supplementary Table S2.

HEK293 cells were cultured in Dulbecco’s modified Eagle’s medium (Sigma) supplemented with 10% FBS (Gibco) and 1% penicillin/streptomycin (Gibco) with 5% CO_2_ at 37 °C. The negative control duplexes and miR-21, miR-378 and miR-133b mimics (double-stranded RNA oligonucleotides) were synthesized by GenePharma. HEK293 cells were co-transfected with pmirGLO-3′UTR-wt plasmid/3′ UTR-edt and the miRNA mimic/negative control. Co-transfection assays were performed in 12-well plates with Lipofectamine 2000 (Invitrogen) according to the manufacturer’s instructions. After transfection for 24 h, cells were harvested. Finally, renilla luciferase and firefly luciferase were detected with the dual-luciferase assay system (Promega).

The effect of a selected A-to-I editing site (chr6:107,039,734) in the 3′-UTR of the *MIB1* gene on *MIB1* expression was evaluated. The mutation in the *MIB1* 3′-UTR was made by site-directed mutagenesis (Takara) and confirmed by Sanger sequencing. The edited and wild-type *MIB1* 3′-UTRs were cloned into the pGL3-basic vector. Luciferase activity and the expression level of firefly luciferase were detected to evaluate the effect of this editing site on *MIB1* mRNA stability.

## 3. Results

### 3.1 Systematic identification of the *S. scrofa* RNA editome in skeletal muscle

To profile the dynamic patterns of RNA editing during skeletal muscle development, we carried out strand-specific RNA-seq in skeletal muscle across 27 developmental stages (SRA accession number SRP158448), including 15 prenatal and 12 postnatal stages, ranging from 33 days after conception to 180 days after birth. A total of 8.2 billion paired-end reads (150 bp × 2) were obtained from 81 transcriptome libraries. A WGS library (SRA accession number SRP157242), which was based on DNA isolated from the ear tissue of an adult male pig with the same genetic background as the pigs from which the samples were collected for RNA-seq, was sequenced. Additionally, our group previously performed WGBS (SRA accession number SRP160645) on the same skeletal muscle samples used for RNA-seq to understand the dynamic regulation of DNA methylation during skeletal muscle development (data not shown). Because differences in DNA and RNA sequence were found to be mainly caused by RNA editing, the WGS and WGBS data were also used in the process of filtering out false-positive DNA-RNA differences caused by heterozygous SNPs. A total of 56.88 billion paired-end reads (150 bp × 2) were obtained from 81 methylome libraries, and 909.92 million paired-end reads (125 bp × 2) were obtained from the WGS library, representing a genome-wide coverage depth of approximately 35× and 47×, respectively. These multi-omic datasets with relatively deep coverage provided an extensive dataset for profiling the RNA editome of developing skeletal muscle.

**Figure 1:**
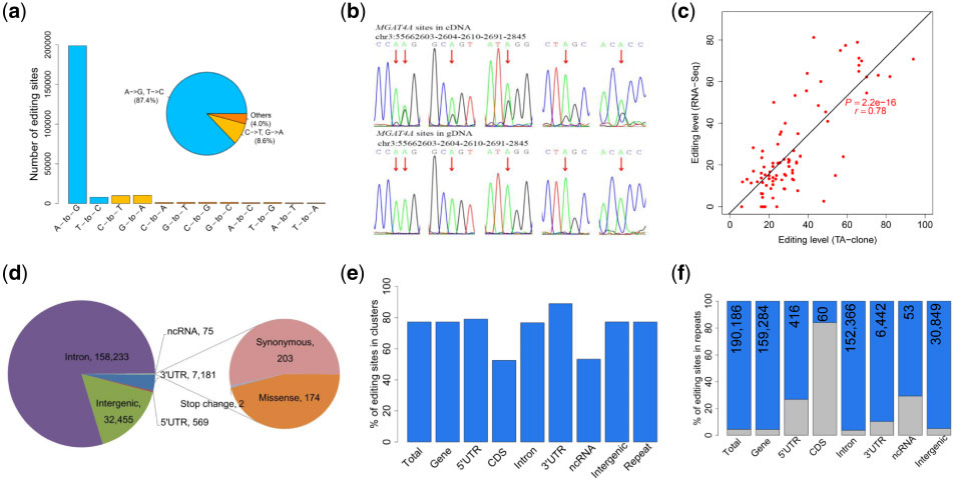
Identification and classification of the *S. scrofa* RNA editome in skeletal muscle. (a) Number and proportions of RNA variant types in pig skeletal muscles. The A-to-G variant, indicating A-to-I editing, is disproportionately enriched. (b) Validation of RNA editing by PCR and Sanger sequencing. For each candidate editing site (indicated by the genome coordinate and a red arrow), raw chromatograms of sequences derived from the cDNA and matched genomic DNA samples are shown. (c) Pearson correlation coefficients (*r*) were calculated to find correlations between editing levels estimated by TA-clonal sequencing and RNA-Seq. (d) Distribution of A-to-I editing sites across different genomic locations. (e) Percentages of A-to-I editing sites occurring in clusters (≥3 sites within a 100-bp window) across different genomic elements. (f) Distribution of A-to-I editing sites in repeat elements. The numbers of A-to-I editing sites in repeat elements across generic regions are shown at the tops of the blue bars. The proportion of A-to-I editing sites not residing in repeat elements is indicated by the grey bar.

Based on the multi-omic dataset mentioned above, we identified 236,569 putative RNA editing sites in skeletal muscle using a pipeline similar to those used in previous studies^2^^,^^32–34^ with slight modifications (Supplementary Fig. S1). We detected four major variant types, including A-to-I (G), T-to-C, G-to-A, and C-to-T editing. Most of these sites (approximately 84%) were produced by A-to-I editing (Fig. 1a). It is noteworthy that C-to-T SNPs in the sample cannot be distinguished from C-to-T substitutions that are caused by bisulphite conversion in WGBS, thus the C-to-U and G-to-A editing sites might be false-positive results and must be filtered from the datasets used in later analyses. Moreover, due to incomplete strand annotation and antisense transcription in the pig reference genome, T-to-C variants could also be considered as possible A-to-I editing sites. Taking these possibilities into consideration, A-to-I and T-to-C editing together accounted for 95.59% of all identified RNA variants (Fig. 1a). Subsequent analyses were mainly focused on A-to-I editing. To validate the RNA editing sites identified based on high-throughput sequencing data, 104 editing sites were randomly selected for experimental confirmation using PCR and Sanger sequencing. The results revealed that 86.5% (90/104) of the selected editing sites were successfully validated (Fig. 1b and Supplementary Fig. S2). These experiments showed that the editing levels calculated by the pipeline used in this study were positively correlated with the editing levels obtained by TA-clonal sequencing (Pearson’s *r* = 0.78, Fig. 1c and Supplementary Table S3). These findings suggest that the identification pipeline is reliable and suitable for use in future studies.

Among A-to-I editing sites, 79.6% of sites were located in intronic regions, followed by intergenic (16.3%) and 3′-UTR (3.6%) regions, while only 379 (0.19%) sites overlapped with protein coding sequences (CDS) (Fig. 1d). A-to-I editing accounted for 58.6% of all RNA variants in the CDS region (Supplementary Fig. S3a). These findings are consistent with reports of depletion of editing sites in the CDS of mammals.^46–48^ Within the editing sites in CDS, 45.9% (174/379) of RNA editing led to changes in amino acids in the encoded proteins (Fig. 1d). Glutamine to arginine (Q-to-R) and threonine to alanine (T-to-A) were the most frequent substitution types (Supplementary Fig. S3b). Meanwhile, the largest fraction of editing sites (77.2%) clearly occurred in clusters on chromosomes. Editing sites in CDS were less likely to be clustered in comparison with editing sites in other genomic elements (Fig. 1e). Moreover, 95.6% of the A-to-I editing sites were found in repeat elements (Fig. 1f). Our data revealed that a substantial proportion of repetitive editing sites (96.2%) were located within SINE/tRNA elements (Supplementary Fig. S4a), although SINEs represented only approximately 10% of the pig genome.^17^ This analysis showed that 67.34% of repetitive editing sites within SINE/tRNA elements were located in the Pre0_SS element, a SINE element of the PRE-1 family (Supplementary Fig. S4b).

### 3.2 Characteristics of A-to-I editing in pig skeletal muscle

**Figure 2:**
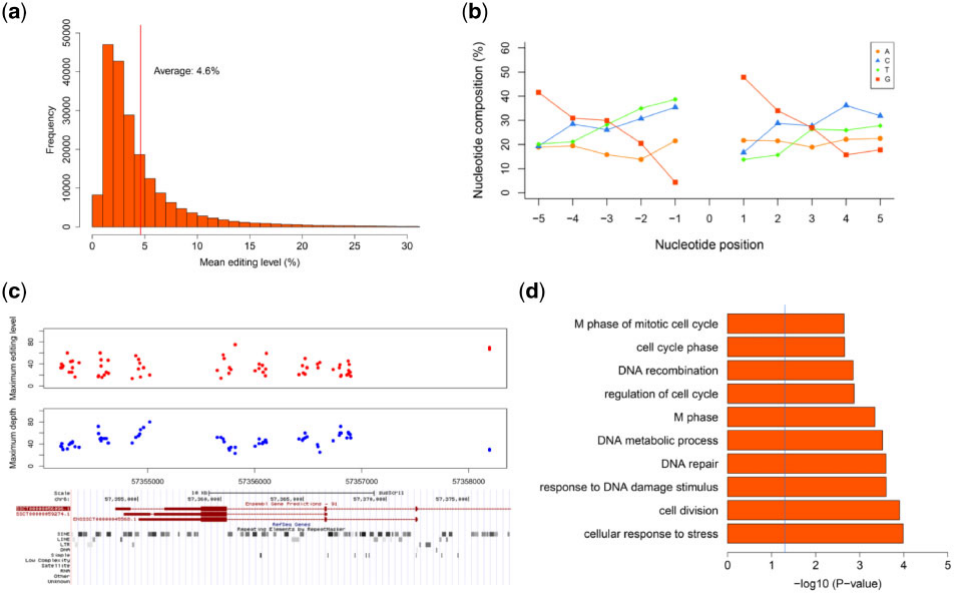
Sequence and structural characteristics of RNA editing sites in pig skeletal muscle. (a) Frequency distribution of the average editing level for all editing sites across skeletal muscle development. The red line is the average editing level of 4.6%. (b) Nucleotide preference flanking the A-to-I editing sites. (c) The gene ENSSSCG00000040433 contained 70 editing sites in its 3′-UTR, in which the editing sites always occurred in clusters. The maximum editing rate (red) and depth (blue) of editing sites and their locations in the genome are shown. (d) GO enrichment analysis of hyper-edited genes that contained at least five editing sites. The top ten biological processes that were reported by DAVID are shown.

In general, RNA editing levels tended to be low at most editing sites in skeletal muscle, with an average editing rate of 4.6% (Fig. 2a), which was consistent previous studies of other mammals.^2^^,^^46^^,^^49^ The sequence and structure around editing sites affected recognition of double-stranded structures by ADARs within their substrates. First, the sequence and structural characteristics of the identified A-to-I editing sites were assessed. As observed in humans,^4^^,^^7^ the sequence around the A-to-I editing sites revealed a strong guanine deficiency one base upstream (∼4.4%) of the editing sites, as well as a strong guanine preference one base downstream (∼47.9%) of the editing sites (Fig. 2b). We also detected a large number of genes with widespread editing sites in exonic regions (490 genes with ≥5 editing sites, Supplementary Fig. S5). The top two hyper-edited genes were ENSSSCG00000040433 and *LAMP2*, which harboured 70 and 45 editing sites, respectively, in their 3′-UTRs (Fig. 2c and Supplementary Fig. S6). These findings may suggest a special regulatory mechanism at the post-transcriptional level for gene expression. GO enrichment analysis revealed that the hyper-edited genes were functionally related to the cell cycle and cell division (Fig. 2d).

Next, conserved A-to-I editing sites across the pig, human, and mouse RNA editomes were identified. In comparison with the A-to-I editing sites in the RADAR and REDIportal databases,^41^^,^^42^ 603 and 39 conserved editing sites between pig and human and between pig and mouse were identified, respectively (Supplementary Fig. S7a). Moreover, 21 of these conserved sites were conserved among the human, mouse, and pig RNA editomes. Next, variation in conserved editing sites across the editomes was assessed among different mouse tissues. In the mouse, RNA-seq dataset from 10 organs and three postnatal skeletal muscles at 0, 20, and 60 days (SRA accession number SRP159202), 42 conserved edited sites were detected. The MDS analysis based on the editing levels of these sites was capable of separating the samples by tissue type (Supplementary Fig. S7b). Tissue profiling revealed that there were higher editing levels and an increase in the number of RNA editing sites in the brain in comparison with other tissues (Supplementary Fig. S7c).

### 3.3 Dynamic changes in A-to-I RNA editing during skeletal muscle development

**Figure 3:**
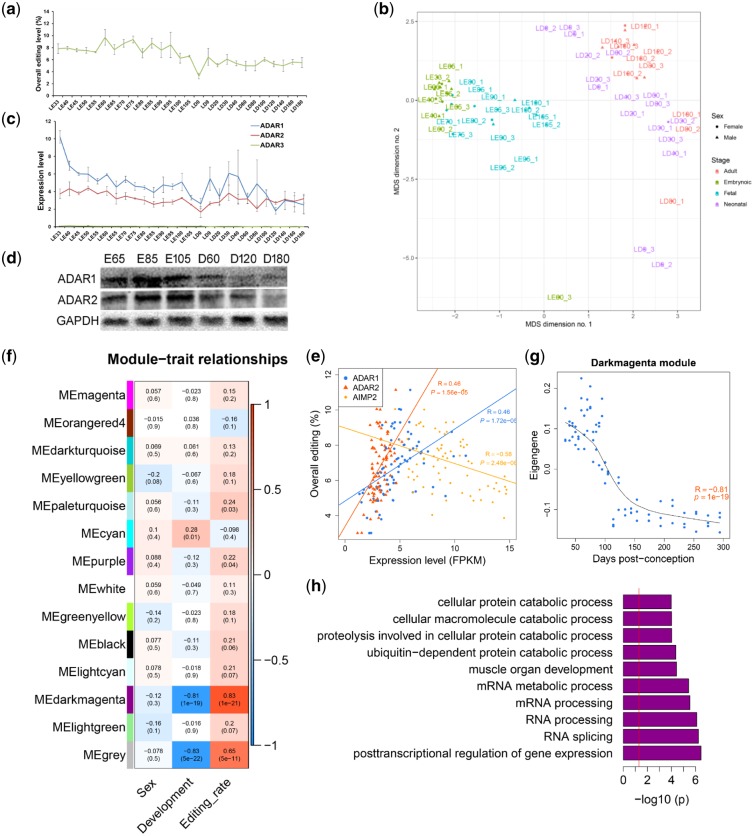
Dynamic landscape of A-to-I editing across skeletal muscle development. (a) Overall editing levels across each stage of skeletal muscle development. The overall editing level of each sample was defined as the total number of reads with G at all candidate editing sites compared with all A and G reads covering the editing sites. (b) MDS analysis of editing levels in skeletal muscles. (c) Expression levels of *ADARs* across skeletal muscle development. In a and c, the error bars shown the standard deviation across three replicates. (d) Western blot analysis of ADAR1 and ADAR2 protein expression during skeletal muscle development. Six representative developmental stages (E65, E85, E105, D60, D120, and D180) were selected. (e) Correlations between the expression levels of *ADAR1/2* and *AIMP2* [quantified as the number of RNA-seq fragments per kilobase of transcript per million mapped reads (FPKM)] and overall editing levels. Pearson correlation coefficients and *P*-values are indicated. (f) Heatmap representing the correlation between module eigenvalue (ME) and the traits of sex, development, and overall editing rate. (g) The eigengene of the ‘darkmagenta’ module is significantly associated with skeletal muscle development. (h) GO enrichment of genes that contain editing sites in the ‘darkmagenta’ module. The top ten biological processes that were reported by DAVID are shown.

The frequency of editing sites and overall editing rate decreased with skeletal muscle development (Fig. 3a and Supplementary Fig. S8). Global levels of RNA editing changed significantly over the course of skeletal muscle development (*r* = −0.61, *P *=* *1.08e−09). The MDS analysis showed that the first dimension explained approximately 10% of the editing variation across all samples, and the prenatal skeletal muscle samples were clearly segregated from the postnatal samples (Fig. 3b). The similar editing status between neighbouring stages reflected gradient changes in RNA editing in skeletal muscle with development. These results suggest that distinct editing profiles exist in prenatal and postnatal skeletal muscles.

During skeletal muscle development, both the mRNA and protein expression levels of *ADAR1* and *ADAR2* were decreased (Fig. 3c and d); however, *ADAR3* was expressed at a low level in all skeletal muscle samples. In addition, we found that the expression levels of both *ADAR1* and *ADAR2* were positively correlated with the overall editing rate (Fig. 3e). *AIMP2* has been reported to be a negative regulator of A-to-I editing.^2^ We found its expression to be higher in skeletal muscle compared with other tissues in both pig (Supplementary Fig. S9a) and mouse (Supplementary Fig. S9b) and more abundant in postnatal muscles than in prenatal skeletal muscles (Supplementary Fig. S9c). There was significantly negative correlation between the expression of *AIMP2* and *ADAR1* (*r* = −0.23, *P *=* *0.03, Supplementary Fig. S9d), and between the overall editing rate and *AIMP2* expression level (*r* = −0.57, *P *=* *2.48e−8, Fig. 3e) during skeletal muscle development.

To identify the key co-editing sites associated with skeletal muscle development, we performed a WGCNA that identified 13 distinct modules of co-editing sites that shared similar editing patterns across skeletal muscle development. We used the eigengenes (the first principal component of each module) to assess the relationship of each module with skeletal muscle development and sex (Fig. 3f). The two largest modules were found to be significantly associated with skeletal muscle development (‘darkmagenta’, *n* = 4,658 sites, *r* = −0.81, *P *=* *1e−19; ‘cyan’, *n* = 1,880 sites, *r* = 0.28, *P *=* *0.01). Notably, the ‘darkmagenta’ module was also positively correlated with the overall editing rate (*r* = 0.83, *P *=* *1e−21) (Fig. 3g). Eight editing sites in transcription factors *MEF2A* and *MEF2C*, which activate many muscle-specific genes during skeletal muscle growth and differentiation,^50^ were included in this module. GO analysis revealed that the ‘darkmagenta’ module was highly enriched for biological processes related to post-transcriptional regulation of gene expression, RNA splicing, and muscle organ development (Fig. 3h). These results reveal a strong correlation between RNA editing and skeletal muscle development.

### 3.4 Differential RNA editing across skeletal muscle development

**Figure 4:**
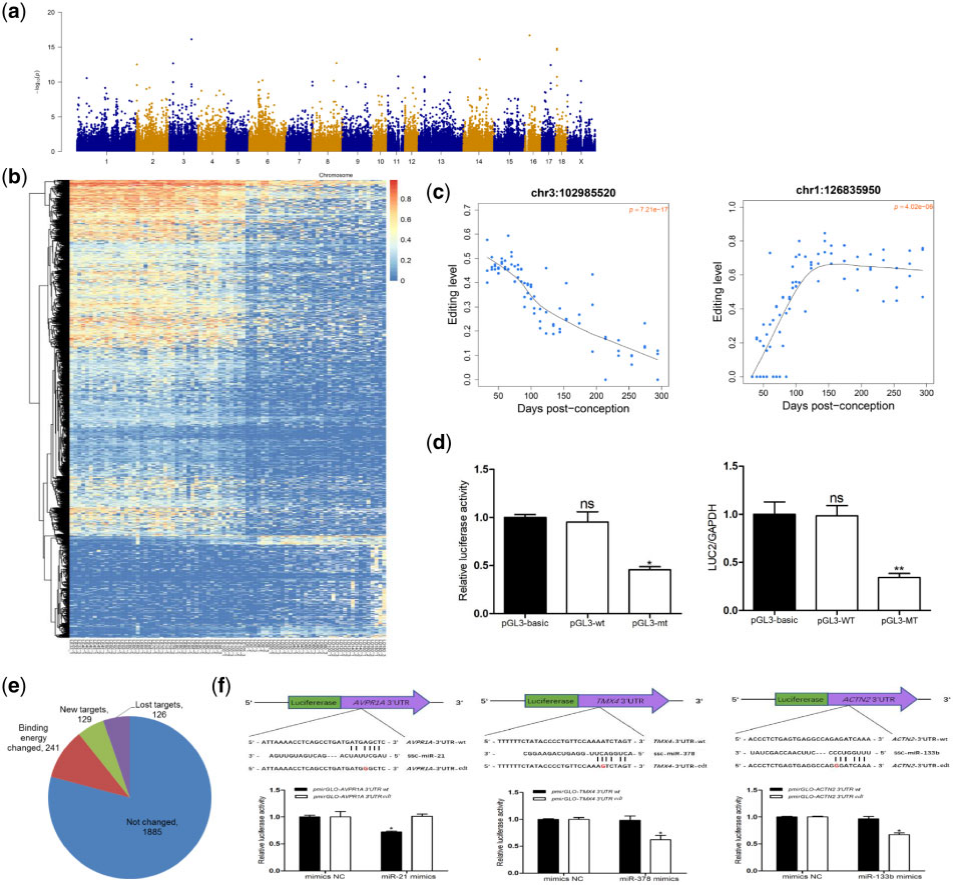
Developmental A-to-I editing patterns and their potential functions during skeletal muscle development. (a) Manhattan plot of the genome-wide *P*-values of association between A-to-I editing and skeletal muscle development. (b) Heatmap showing the editing profiles of dDESs across skeletal muscle development. (c) Two representative dDESs showing a trend for decreasing or increasing editing across skeletal muscle development. (d) Validation of the effect of an A-to-I editing site (chr6:107,039,734) on the mRNA expression of its host gene. Luciferase activity (*left*) and the expression level (*right*) of firefly luciferase were detected to evaluate the effect of this editing site on *MIB1* mRNA stability. Ns, no significant difference (*P *> 0.05), *P <* *0.05, **P <* *0.01. (e) Distribution of editing sites related to miRNA target sites in the 3′-UTR and possible consequence of RNA editing. (f) Validation of the effects of A-to-I editing on miRNA-target binding using a dual luciferase reporter assay. Three editing sites (chr5:27,827,411, chr17:16,833,004, and chr14:54,710,988) in the 3′-UTRs of *AVPR1A* (*left*), *TMX4* (*middle*), and *ACTN2* (*right*), respectively, were randomly selected. Schematics of the predicted binding sites between miRNAs and the 3′-UTRs of the target genes are shown (*upper*). The positions of the editing sites are indicated in red. HEK293 cells were co-transfected with luciferase reporters carrying the wild-type or edited-type 3′-UTRs of *AVPR1A*, *TMX4*, and *ACTN2*, as well as their corresponding miRNA mimic/negative control duplexes. Relative luciferase activity was measured after 24 h (*lower*). The data are represented as mean ± SEM (*n* = 3). *P <* *0.05.

We performed multiple linear regression analysis to search for developmentally differential editing sites (dDESs) across skeletal muscle development. A total of 1,608 sites (0.95% of the 169,661 sites, FDR <0.05) showed significant editing rate differences (Fig. 4a and b, Supplementary Table S4). These dDESs were edited in the majority of the 81 tested skeletal muscle samples (average *n* = 42.5, Supplementary Fig. S10a). The top ranked up-regulated and down-regulated dDESs are shown in Fig. 4c. The distribution ratio of dDESs was relatively consistent across autosomal and X chromosomes (Supplementary Fig. S10b). Two chromosomes showed significant enrichment of dDESs; 1.18% and 1.15% of editing sites were identified as significant dDESs in chromosome 6 (relative enrichment = 1.266, *P *=* *0.002) and chromosome 3 (relative enrichment = 1.233, *P *=* *0.022), respectively (Table 1). Additionally, dDESs were not equally distributed in different genic features. dDESs were significantly enriched in the 3′-UTR (3.81%, relative enrichment = 4.14, *P *=* *8.58e−72) and 5′-UTR (4.50%, relative enrichment = 4.92, *P *=* *3.97e−09). In contrast, dDESs were underrepresented in intronic regions (0.78%, relative enrichment = 0.82, *P *=* *3.05e−07) (Supplementary Fig. S10c, Table 2). GO analysis revealed that genes harbouring at least one dDES were significantly associated with protein amino acid phosphorylation, muscle organ development and RNA splicing (Supplementary Fig. S10d). Of note, several dDESs were located in genes that play key roles in skeletal muscle development, including muscle-specific gene *MEF2A*, which controls cell growth, survival and apoptosis in skeletal muscle; skeletal muscle contractile gene *MYH3*; and *SGCA*, which affects fast-twitch and slow-twitch skeletal muscles.^51^ These results imply that RNA editing exhibited stage-specific patterns and might play an important role during skeletal muscle development. Additionally, no sex-bias editing sites reached the significance threshold of FDR ≤0.05 (Supplementary Fig. S11), implying that development was the major contributor to editing variation in skeletal muscle.

**Table 1:** Distribution of dDESs significantly associated with skeletal muscle development in different chromosomes

Chr	Total sites	Significant sites (%)	Enrichment (95% CI)	*P*-value
1	17,918	146 (0.815)	0.869 (0.728–1.031)	0.109
2	12,029	129 (1.072)	1.146 (0.95–1.374)	0.143
3	11,626	134 (1.153)	**1.233** (1.025–1.473)	**0.023**
4	9,151	102 (1.115)	1.192 (0.965–1.459)	0.096
5	7,840	66 (0.842)	0.898 (0.69–1.15)	0.434
6	16,396	194 (1.183)	**1.266** (1.084–1.472)	**0.002**
7	8,571	68 (0.793)	0.846 (0.653–1.08)	0.185
8	7,564	72 (0.952)	1.016 (0.79–1.289)	0.855
9	8,585	80 (0.932)	0.995 (0.784–1.247)	1.000
10	5,034	34 (0.675)	0.719 (0.496–1.011)	0.062
11	4,070	46 (1.13)	1.209 (0.88–1.624)	0.217
12	7,106	53 (0.746)	0.795 (0.592–1.046)	0.114
13	16,601	134 (0.807)	0.861 (0.716–1.028)	0.098
14	11,778	102 (0.875)	0.933 (0.756–1.14)	0.552
15	8,635	77 (0.892)	0.952 (0.746–1.198)	0.731
16	3,993	36 (0.902)	0.962 (0.67–1.34)	0.934
17	4,167	44 (1.056)	1.129 (0.815–1.525)	0.416
18	3,695	24 (0.65)	0.691 (0.441–1.034)	0.082
X	3,388	33 (0.974)	1.04 (0.712–1.47)	0.787

Note: Significant P values (< 0.05) and corresponding enrichment were marked in boldface.

**Table 2:** Distribution of dDESs significantly associated with skeletal muscle development in different genic features

Genic feature	Total sites	Significant sites (%)	Enrichment (95% CI)	*P*-value
3′-UTR	6,934	264 (3.807)	**4.137** (3.609–4.726)	**8.58e−72**
5′-UTR	489	22 (4.499)	**4.923** (3.047–7.57)	**3.97e−09**
intergenic	24,477	250 (1.021)	1.078 (0.939–1.234)	0.276
intron	137,418	1066 (0.776)	**0.817** (0.755–0.884)	**3.05e−07**
CDS	343	6 (1.749)	1.861 (0.677–4.102)	0.149

### 3.5 Functional implications of dDESs in developing skeletal muscle

We further explored the functional implications of the identified dDESs in skeletal muscle development. Only five dDESs were located in CDS regions, and four of these dDESs caused non-synonymous shifts, which were located in the *DUSP11*, *DACT3*, *SACS*, and *CDK13* genes. DUSP11 negatively regulates members of the mitogen-activated protein (MAP) kinase superfamily associated with cellular proliferation and differentiation, and DACT3 is a key regulator of canonical and/or non-canonical Wnt signalling pathways during development.^52^ The editing level of the dDES (chr18:54,429,632) in the CDS of *CDK13* were decreased during skeletal muscle development (Supplementary Fig. S10e). This gene is a member of the cyclin-dependent serine/threonine protein kinase family and regulates cell-cycle progression and gene expression.^53^

We next investigated the correlations between the editing rates of genic dDESs and the abundance of their host mRNAs. Most of the identified dDESs were positively or negatively correlated with host mRNA levels. The distribution of correlation coefficients was bimodal, and most dDESs exhibited significant positive or negative correlation with host mRNAs (Supplementary Fig. S12a). For example, the editing level of editing site chr6:107,039,734, which is in the 3′-UTR of *MIB1*, was negatively correlated with *MIB1* expression (*r* = −0.75, *P *=* *4.73e−16). Our experiments validated that both luciferase activity and the expression level of firefly luciferase for the editing type were significantly lower than those associated with the wild type, confirming that this editing site significantly affected the expression of its host gene (Fig. 4d). However, no bimodal distribution of correlation coefficients was observed between all editing sites and their host gene expression levels (Supplementary Fig. S12b). Additionally, the correlation coefficients for 3′-UTR dDESs were significantly different from those of dDESs in intronic regions (Supplementary Fig. S12c), suggesting that dDESs in 3′UTRs have *cis*-regulatory potential that could impact gene expression by affecting stabilization or degradation of mRNAs.

A functional relationship between RNA-editing and miRNA mediated post-transcriptional gene silencing has been reported.^14^^,^^37^^,^^48^^,^^54^ The binding energy between miRNA and target regions around the editing sites was computationally predicted. This analysis showed that 3′-UTRs with A-to-I editing had miRNA binding energies lower than those of 3′-UTRs without RNA editing (Welch’s *t*-test, *t*_19,__405_ = −16.477, *P *<* *2.2e−16) (Supplementary Fig. S13). To explore the potential negative impacts of dDESs on miRNA binding, we first determined whether dDESs in 3′-UTRs (264 sites) could be targeted by miRNAs. A total of 2,381 possible miRNA-3′UTR interaction pairs were predicted, among which 241 miRNA-3′-UTR pairs exhibited changes in binding energy (147 sites). Moreover, 88 editing sites may create miRNA binding sites with the potential to generate 129 new miRNA-3′-UTR interaction pairs. Additionally, 91 of the editing sites led to a disruption of miRNA recognition, which resulted in the loss of 126 possible miRNA-3′-UTR interactions (Fig. 4e). Three editing sites (chr5:27,827,411, chr17:16,833,004, and chr14:54,710,988) in the 3′-UTRs of *AVPR1A*, *TMX4*, and *ACTN2* were selected for validation of their interactions with miRNAs. The dual luciferase reporter assay successfully validated the influence of RNA editing on the binding of miRNAs to mRNAs. As shown in Fig. 4f, miR-21 markedly decreased the luciferase activity of wild-type *AVPR1A* by binding to its 3′-UTR, while the edited form of *AVPR1A* was not susceptible to this effect (Fig. 4f). In comparison with the wild-type forms, the luciferase activities of *TMX4* and *ACTN2* with RNA editing in their 3′-UTRs were significantly decreased by miR-378 and miR-133b, respectively (Fig. 4f). These findings demonstrate that RNA editing regulates gene expression by affecting miRNA-mRNA interaction in skeletal muscle.

### 3.6 Database of *S. scrofa* A-to-I editome

**Figure 5:**
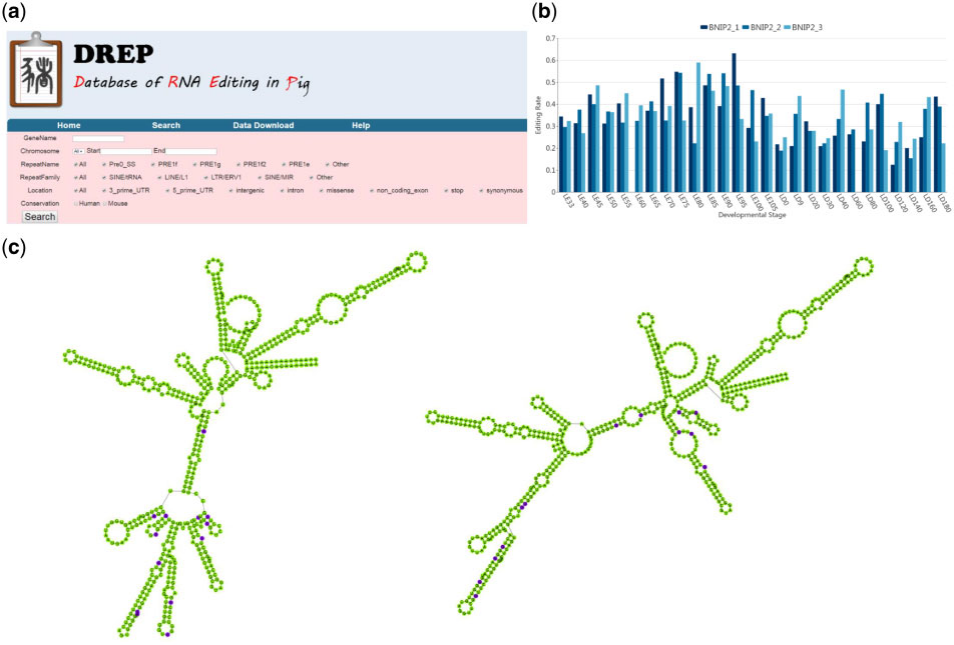
Database for *S. scrofa* A-to-I RNA editing in skeletal muscle. (a) The DREP search page. (b) A representative graph showing the editing level of an editing site (chr1:112,369,117) during skeletal muscle development. (c) Representative graphs showing the secondary structures of the reference (left) and edited (right) sequences. The editing sites were marked in purple.

Finally, we developed a user-friendly and free database [the Database of RNA Editing in Pig (DREP)] that allows researchers to retrieve information about the A-to-I editing sites identified in the present study (http://www.rnanet.org/editing/home.html). Users can use the query interface and web interface of DREP to search RNA editing annotations, including their locations in the genome, genes, genic components (missense, synonymous, 5′-UTR, 3′-UTR, ncRNA, intronic, intergenic), repetitive elements (repetitive, non-repetitive), repetitive element family membership (Fig. 5a) and editing level in skeletal muscle across 27 development stages (Fig. 5b), as well as editing conservation across human and mouse. The locations of the editing sites in the genome can be seen using the Jbrowser. If an editing site located in the 3′-UTR and is targeted by miRNAs, the miRNA binding information is exhibited. The secondary structures of reference sequences and A-to-I editing sequences could also been seen in the website (Fig. 5c). To facilitate more detailed searches, DREP also provides the entire editing database contents as flat files for free download. This database serves as an informative and valuable data source for the study of RNA editing in mammalian skeletal muscle.

## 4. Discussion

With the rapid adoption of genome re-sequencing and RNA-seq technologies, a large number of RNA editing sites in the genome have been identified in various species.^2–7^ However, RNA editing in developing skeletal muscle remains poorly understood. In this study, we carried out a comprehensive profiling of *S. scrofa* RNA editing in skeletal muscle across 27 developmental stages. Using multiple-omics datasets with high sequencing depth and coverage, including WGS, WGBS, and strand-specific RNA-seq data, we captured unprecedented editing events with low editing levels and in low depth regions. To our knowledge, this is the first systematic study of RNA editing in mammalian skeletal muscle.

We detected a total of 236,569 RNA editing sites. Consistent with observations of RNA editing in other mammals,^2^^,^^4^^,^^46^ A-to-I editing appears to be the dominant form and accounted for 84% of all identified sites. The majority of the A-to-I editing sites occur in clusters, repetitive elements, and non-coding regions. In primates, A-to-I editing primarily occurs within Alu repeat elements,^2^^,^^9^ which are primate-specific SINEs. The pig genome is devoid of Alu repeat elements; thus, we confirmed that the majority of A-to-I editing occurred within SINE/tRNA elements in pig.^26^ Additionally, depletion of editing events and a reduced tendency towards clusters and editing site repeat elements were observed in CDS regions.^46–48^ The neighbour sequence preferences could be considered as a potential *cis*-regulatory mechanism.^48^ Our study suggested that the editing site had a strong aversion to G upstream of the editing site, while it had a preference for G downstream of the editing site; this preference agreed with the known substrate recognition characteristics of *ADARs*.^55^^,^^56^ A set of homologous editing sites was conserved across the human, mouse and pig editosomes, but more than half of the conserved sites in mice are also edited in humans. Additionally, 42 homologous sites in pig skeletal muscle were conserved and edited in mouse tissues, and none of these sites were edited in mouse postnatal skeletal muscles, confirming that RNA editing exhibits a high degree of spatiotemporal specificity.

Our previous studies revealed that higher transcriptional activity and more complex molecular events occurred in prenatal skeletal muscles compared with postnatal skeletal muscles (data not published). We found that both the frequency and level of RNA editing were decreased across skeletal muscle development, which is contrary to observations in the brain.^48^^,^^57^ Previous studies have shown that RNA editing plays an important role in various developmental processes,^12–14^ but its role in skeletal muscle development has not been explored. ADAR family proteins constitute the key enzymatic activity for A-to-I editing. In mammals, ADAR1 and ADAR2 exhibits ubiquitous expression and are catalytically active, while ADAR3 is considered to be inactive.^8^ A previous study revealed that ADAR1 represses myotube maturation by targeting and modulating the expression of certain myogenesis-associated genes.^25^ Our results show that expression of *ADAR1* underwent stage-specific alterations; the mRNA and protein levels of ADAR1/2 were down-regulated during skeletal muscle development, while ADAR3 was not expressed in skeletal muscle. In addition, we found that the overall editing rate exhibited a significant positive correlation with the level of *ADAR1/2* expression during skeletal muscle development. These results suggest that A-to-I RNA editing mediated by ADAR1/2 is involved in myogenesis.

We detected thousands of dDESs and noted that the editing levels of most dDESs were decreased during skeletal muscle development. Additionally, most dDESs were significantly correlated with host mRNA levels and functionally associated with muscle organ development. Moreover, we experimentally validated the effects of RNA editing on host gene expression *in vitro*. These experiments confirm the functionality of these editing sites in skeletal muscle development. Previous studies have suggested that RNA editing at 3′-UTRs is developmentally regulated^58^ and might disrupt miRNA-mediated post-transcriptional gene silencing and gene expression.^14^^,^^59^ Remarkably, we noted that dDESs were considerably enriched at the 3′-UTR and confirmed that dDESs at the 3′-UTR might affect miRNA target binding. These results suggest that A-to-I editing may play a regulatory role during skeletal muscle development. Recent advances in RNA-editing research have led to the creation of several RNA editing resources for humans, mice, and flies.^41^^,^^42^^,^^60^^,^^61^ Although each of these resources provide valuable information, a database of RNA editing in the pig is currently lacking. Our present database provides information regarding the dynamic landscape of RNA editing during pig skeletal muscle development, which might be useful for researchers who study skeletal muscle development and animal breeders developing new molecular markers for meat production traits.

In summary, we created a comprehensive and dynamic atlas of the *S. scrofa* RNA editome in skeletal muscle across 27 developmental stages. To our knowledge, this is the first genome-wide atlas of the RNA editome of developing animal skeletal muscle. This work identified a series of RNA editing sites associated with skeletal muscle development and revealed their potential roles in myogenesis. This study presents new insight into skeletal muscle development and provides abundant information regarding regulation of RNA editing. This knowledge may be helpful to animal breeders, as well as to biomedical researchers studying muscle-related diseases.

## Availability of data

Sequencing data have been deposited to Sequence Read Archive (SRA) at the National Center for Biotechnology Information (NCBI) under accession numbers SRP157242, SRP160645, SRP158448 and SRP159202.

## Supplementary Material

dsz006_Supplementary_Data
