# Supplementary material for: Developmental atlas of the RNA editome in Sus scrofa skeletal muscle
Source: DNA Res. 2019 Apr 23;26(3):261–72. doi: 10.1093/dnares/dsz006 (PMC6589548; doi:10.1093/dnares/dsz006)
Supplement: dsz006_Supplementary_Data [file dsz006_supplementary_data.zip › dsz006-Suppl_data/Supplementary Figures.pdf]

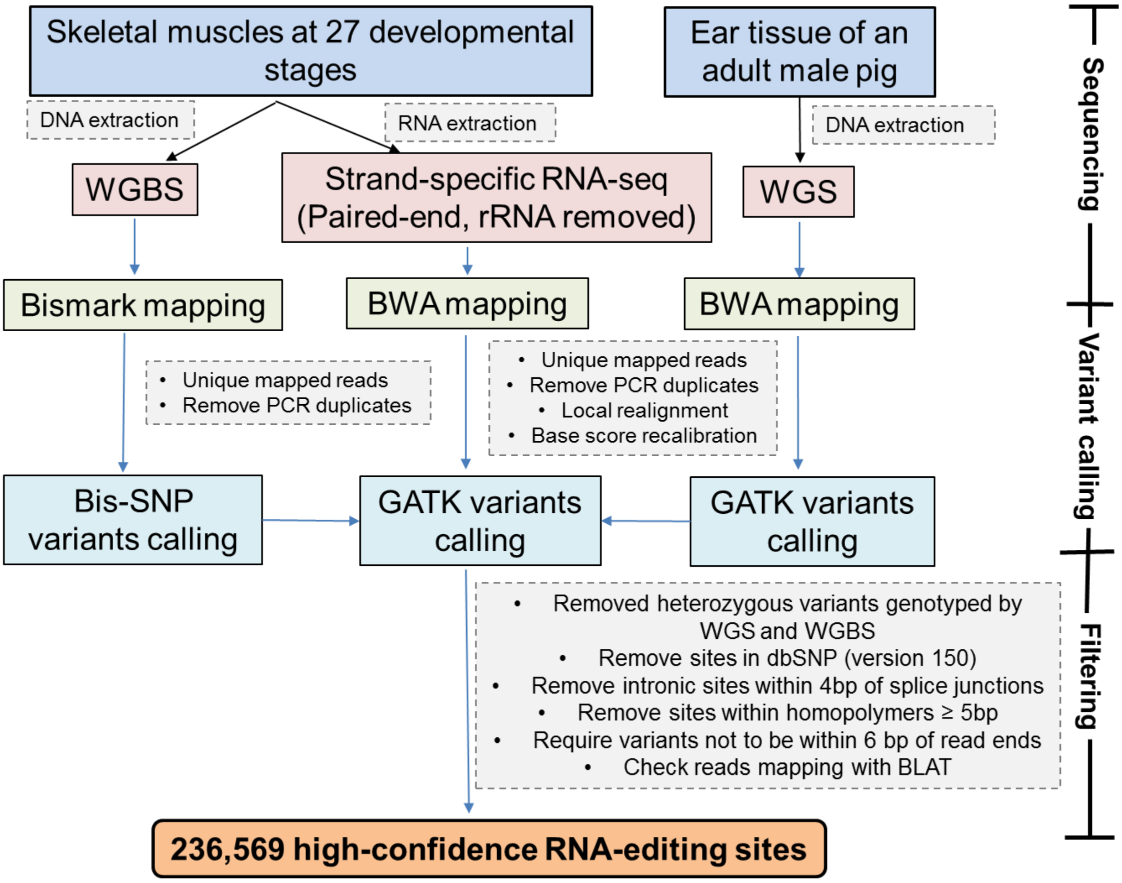

Supplementary Figure S1. Overview of the pipeline of RNA editing identification. Whole genome sequencing, genome-wide bisulfite sequencing and strand-specific rRNA-depleted total RNA sequencing data were used to identify RNA editing in pig skeletal muscle. Multiple filters were applied to control false positives caused by library construction, sequencing, and misalignment.

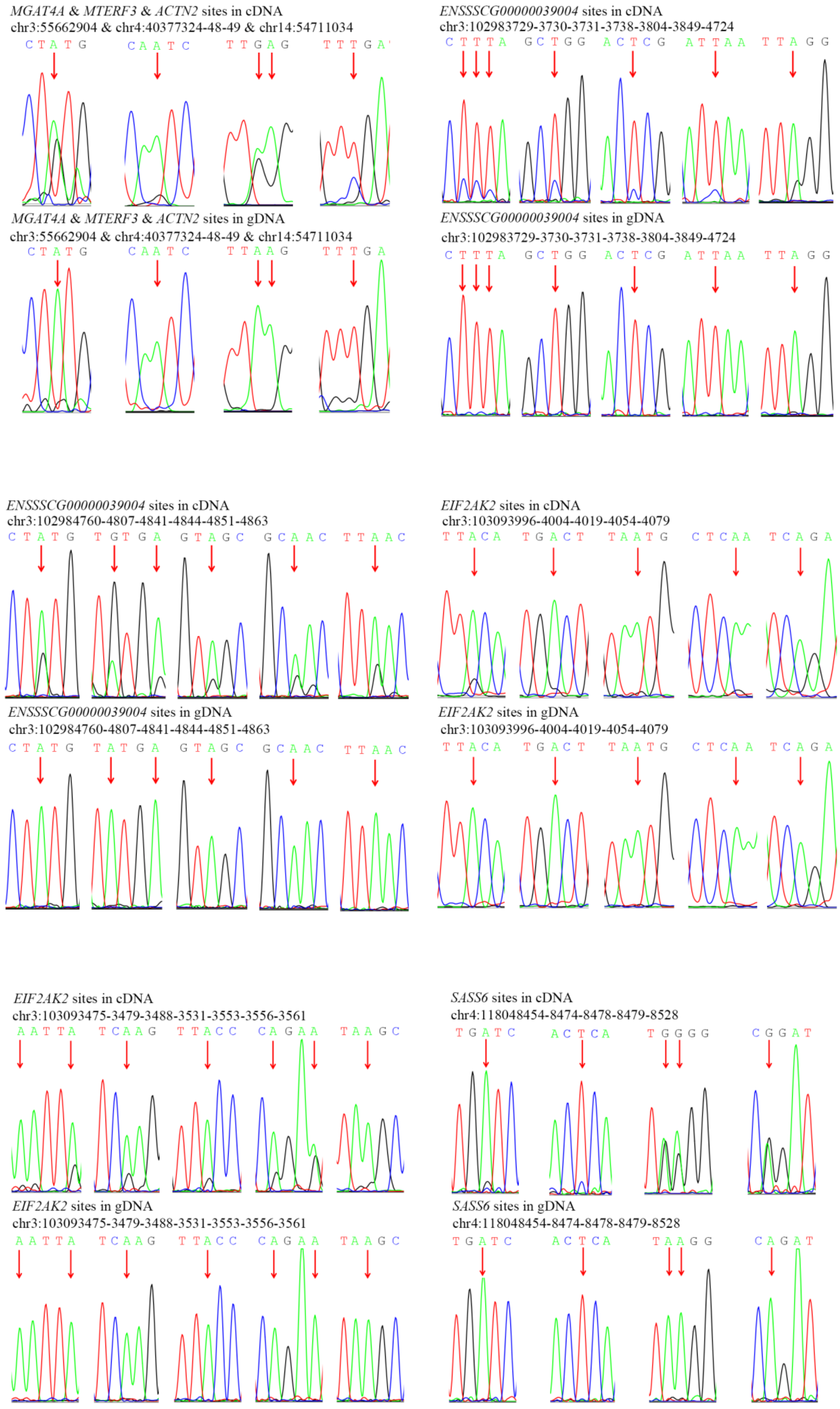
MGAT4A & MTERF3 & ACTN2 sites in cDNA
chr3:55662904 & chr4:40377324-48-49 & chr14:54711034
C T A T G
C A A T C
T T G A G
T T T G A
MGAT4A & MTERF3 & ACTN2 sites in gDNA
chr3:55662904 & chr4:40377324-48-49 & chr14:54711034
C T A T G
C A A T C
T T A A G
T T T G A
ENSSSCG00000039004 sites in cDNA
chr3:102983729-3730-3731-3738-3804-3849-4724
C T T T A
G C T G G
A C T C G
A T T A A
T T A G G
ENSSSCG00000039004 sites in gDNA
chr3:102983729-3730-3731-3738-3804-3849-4724
C T T T A
G C T G G
A C T C G
A T T A A
T T A G G
ENSSSCG00000039004 sites in cDNA
chr3:102984760-4807-4841-4844-4851-4863
C T A T G
T G T G A
G T A G C
G C A A C
T T A A C
ENSSSCG00000039004 sites in gDNA
chr3:102984760-4807-4841-4844-4851-4863
C T A T G
T A T G A
G T A G C
G C A A C
T T A A C
EIF2AK2 sites in cDNA
chr3:103093996-4004-4019-4054-4079
T T A C A
T G A C T
T A A T G
C T C A A
T C A G A
EIF2AK2 sites in gDNA
chr3:103093996-4004-4019-4054-4079
T T A C A
T G A C T
T A A T G
C T C A A
T C A G A
EIF2AK2 sites in cDNA
chr3:103093475-3479-3488-3531-3553-3556-3561
A A T T A
T C A A G
T T A C C
C A G A A
T A A G C
EIF2AK2 sites in gDNA
chr3:103093475-3479-3488-3531-3553-3556-3561
A A T T A
T C A A G
T T A C C
C A G A A
T A A G C
SASS6 sites in cDNA
chr4:118048454-8474-8478-8479-8528
T G A T C
A C T C A
T G G G G
C G G A T
SASS6 sites in gDNA
chr4:118048454-8474-8478-8479-8528
T G A T C
A C T C A
T A A G G
C A G A T

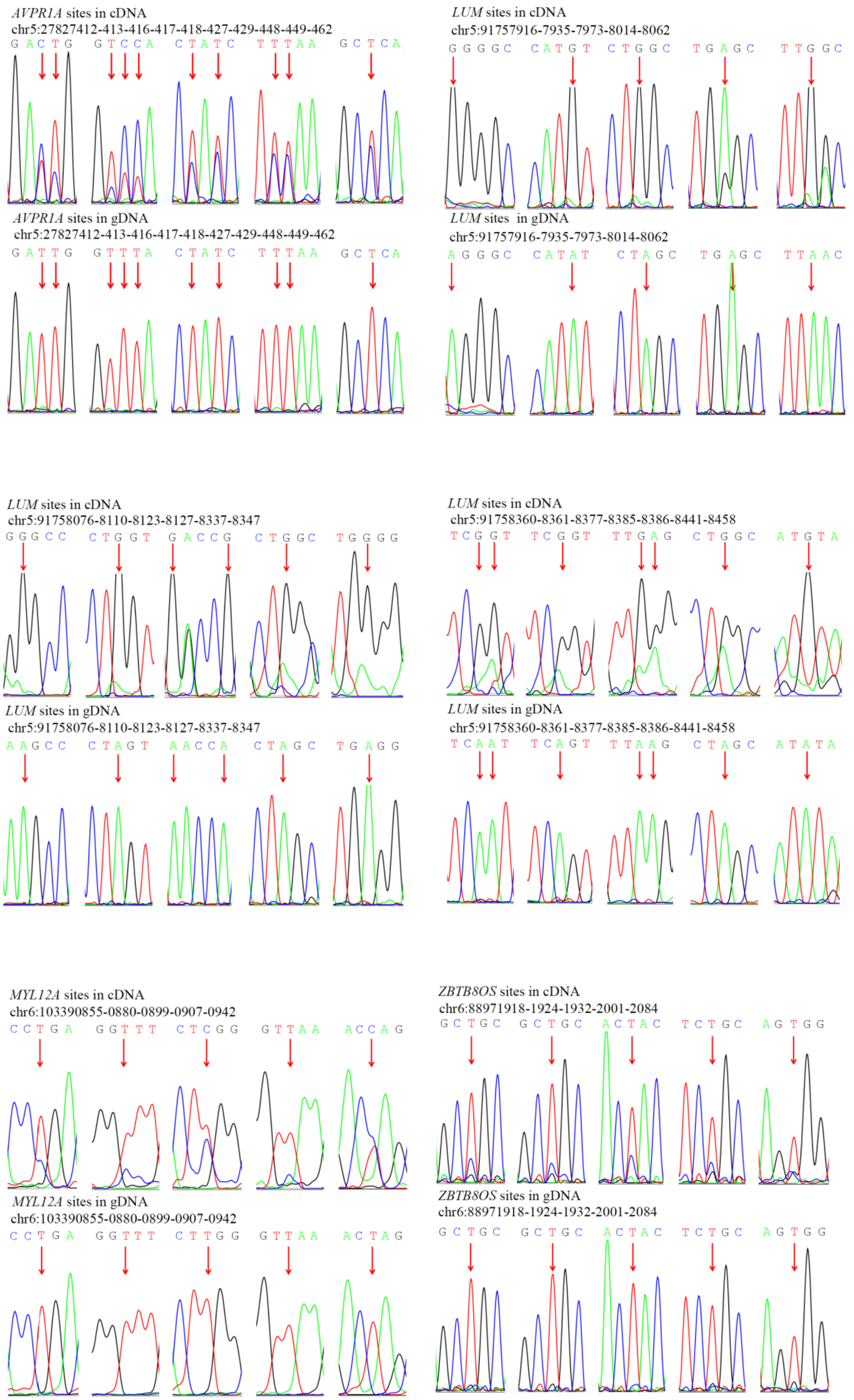
AVPR1A sites in cDNA
chr5:27827412-413-416-417-418-427-429-448-449-462
AVPR1A sites in gDNA
chr5:27827412-413-416-417-418-427-429-448-449-462
LUM sites in cDNA
chr5:91757916-7935-7973-8014-8062
LUM sites in gDNA
chr5:91757916-7935-7973-8014-8062
LUM sites in cDNA
chr5:91758076-8110-8123-8127-8337-8347
LUM sites in gDNA
chr5:91758076-8110-8123-8127-8337-8347
LUM sites in cDNA
chr5:91758360-8361-8377-8385-8386-8441-8458
LUM sites in gDNA
chr5:91758360-8361-8377-8385-8386-8441-8458
MYL12A sites in cDNA
chr6:103390855-0880-0899-0907-0942
MYL12A sites in gDNA
chr6:103390855-0880-0899-0907-0942
ZBTB8OS sites in cDNA
chr6:88971918-1924-1932-2001-2084
ZBTB8OS sites in gDNA
chr6:88971918-1924-1932-2001-2084

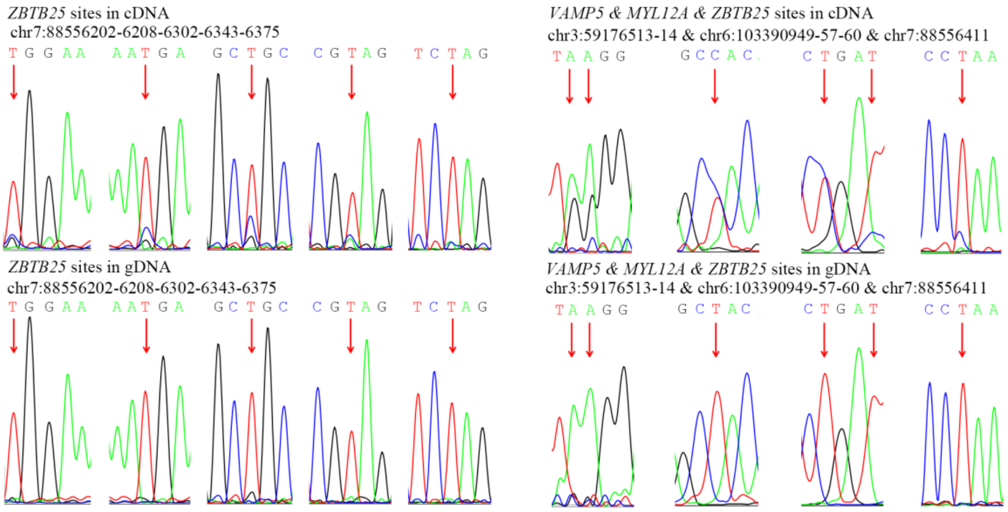

Supplementary Figure S2. Validation of RNA editing by PCR and Sanger sequencing. For each candidate editing site (indicated by genome coordinate and red arrow), raw chromatograms of sequences derived from the cDNA and matched genomic DNA samples are shown. Overall, 90 candidate editing sites are successfully validated.

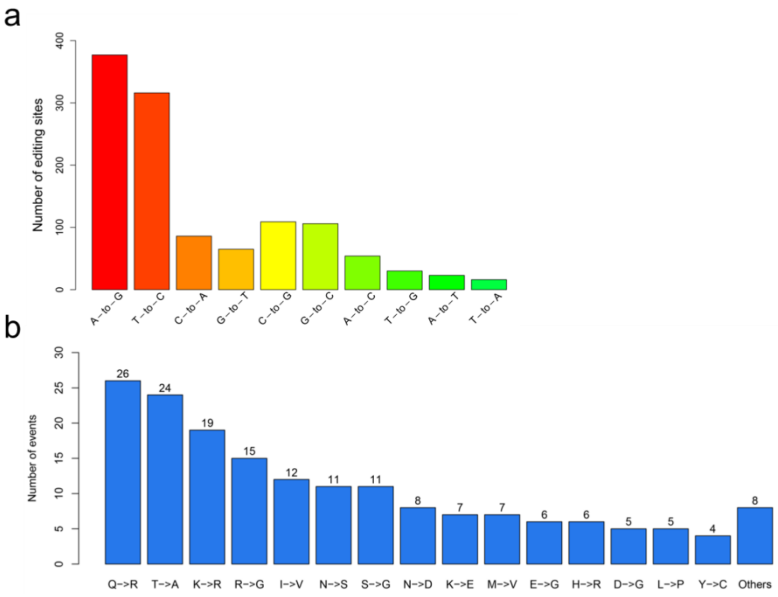

Supplementary Figure S3. A-to-I editing in CDS region. (**a**) Number of RNA variant types in CDS region. Because C-to-T substitutions might be false positives caused by bisulfite conversion in WGBS, the C-to-U and G-to-A editing sites were excluded. The two editing types (A-to-I and T-to-C) together accounted for 58.6% of all RNA variants in CDS. (**b**) Distribution of amino acid changes caused by missense editing.

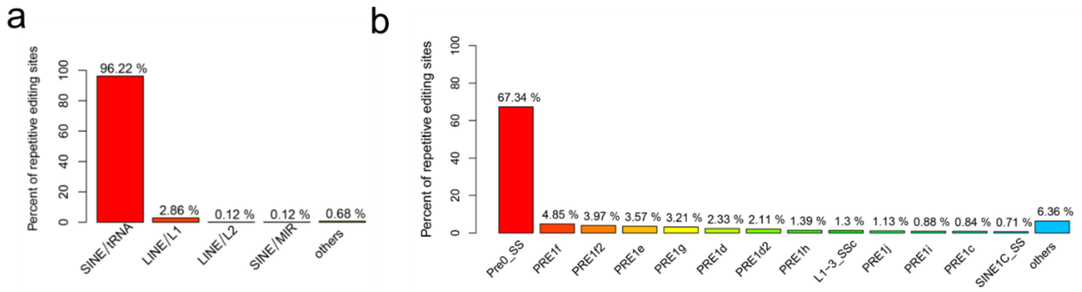

Supplementary Figure 4. Distribution of repetitive A-to-I editing sites across major repetitive element families (**a**) repetitive element types across (**b**).

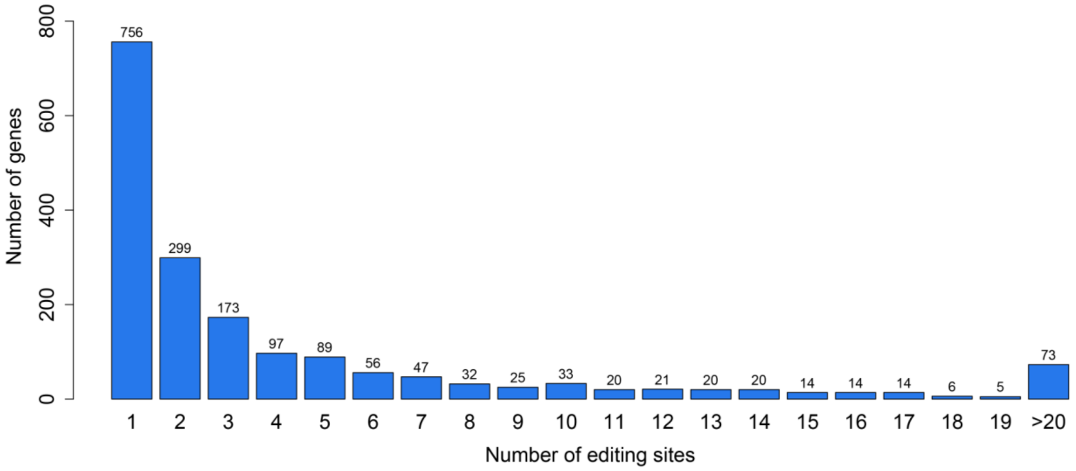

Supplementary Figure S5. Statistic for numbers of editing sites per gene.

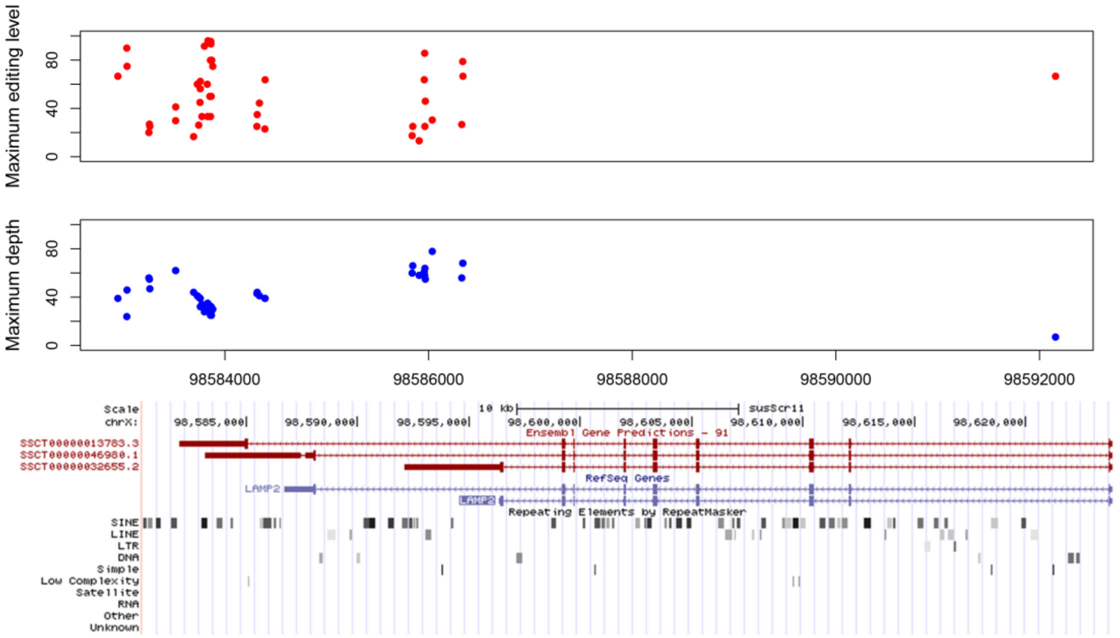

Supplementary Figure S6. The gene LAMP2 contained 45 editing sites in its 3′UTR. Maximum editing rate (red) and depth (blue) of editing sites, and their location in the genome are shown.

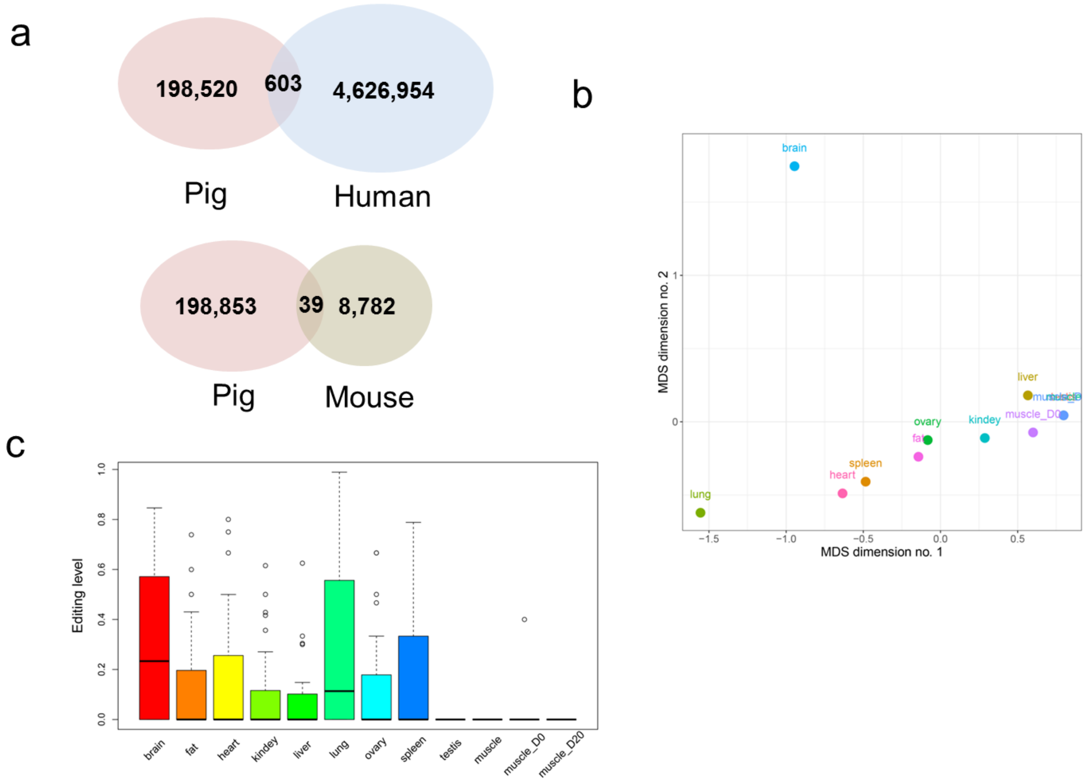

Supplementary Figure S7. Conservation analysis of RNA editing across human, mouse and pig. (**a**) Venn diagram showing the number of conserved editing sites between pig and human (*Upper*) and between pig and mouse (*Lower*). The human and mouse A-to-I editing sites were downloaded from RADAR (v2, http://rnaedit.com/) and REDIportal databases.[1,2] (**b**) MDS of editing profiles in various mouse tissues. The samples were largely separated by tissue type. (**c**) Boxplot showing edditing levels of the conserved and edited sites in various mouse tissues.

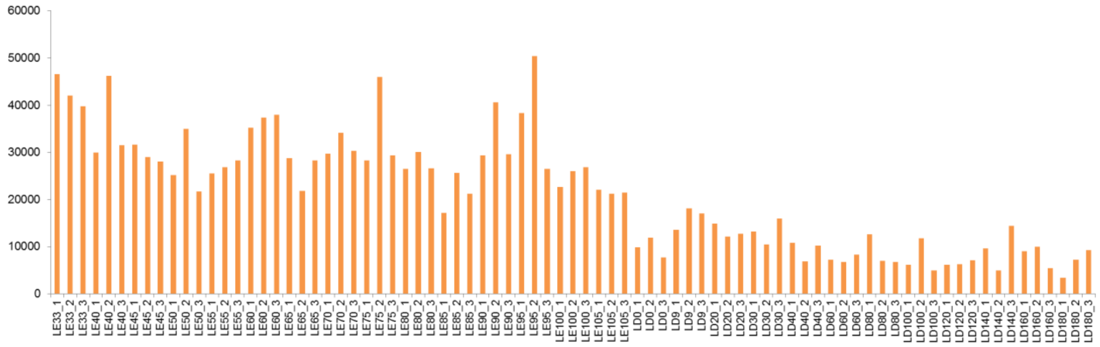

Supplementary Figure S8. Number of editing sites in the 81 skeletal muscles.

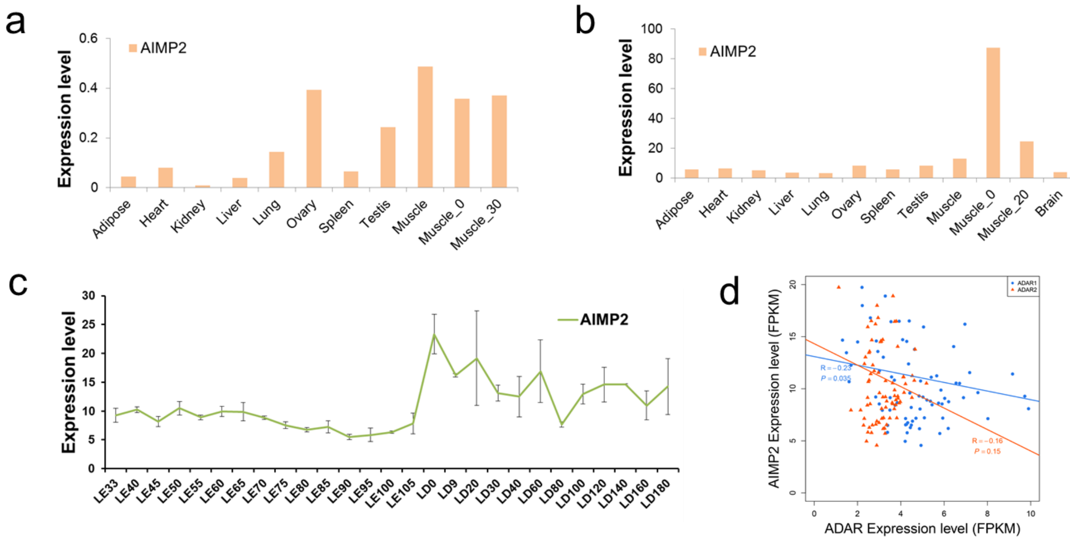

Supplementary Figure S9. Expression of *AIMP2* gene. (**a**) Expression levels of the ADAR gene in different tissues of pigs based on our previous RNA-seq studies.[3,4] (**b**) Expression levels of the *AIMP2* gene in different mouse tissues based on RNA-seq data which were deposited to Gene Expression Omnibus under the accession number SRP159202. (**c**) Expression levels of *AIMP2* at 27 developmental stages of skeletal muscle. Error bars are standard deviations (SD) across three replicates. (**d**) Correlations between expression levels of *ADAR1/2* and *AIMP2* during skeletal muscle development. The Pearson correlation coefficient and *P* value are indicated.

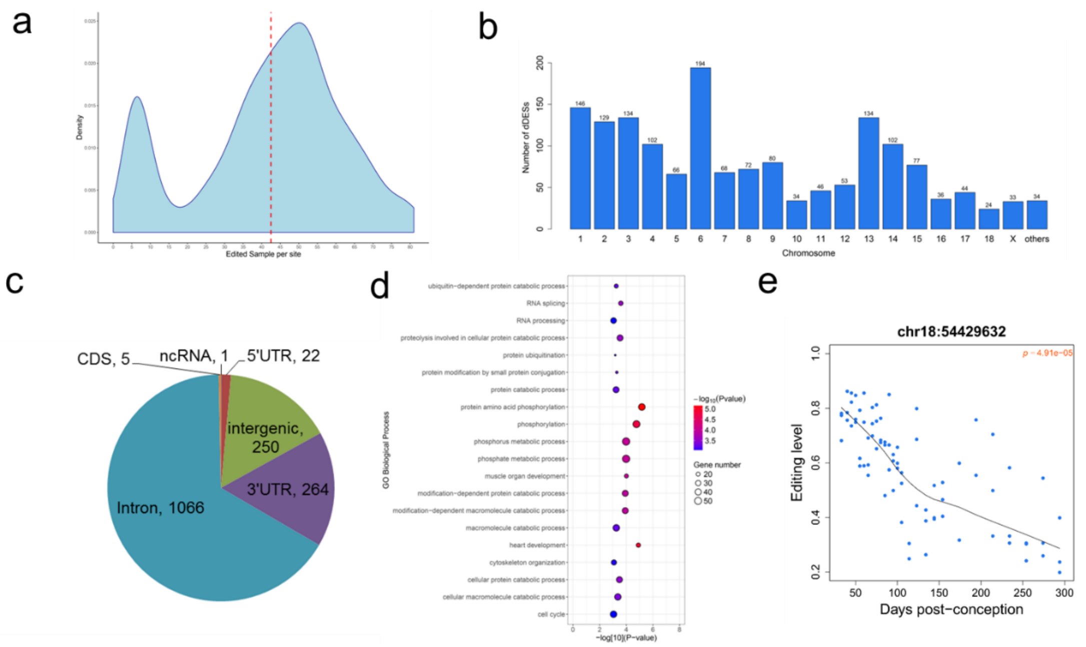

Supplementary Figure S10. Characteristic of the dDESs. (**a**) Distribution of the number of edited samples per editing site. The mean value (red dashed line) is indicated. Distribution of dDESs across different chromosomes (**b**) and across different genomic loations (**c**). (**d**) GO enrichment analysis of genes with dDESs. The top 20 biological processes reported by DAVID are shown. (**e**) The editing level of a dDES (chr18:54,429,632) in the CDS of *CDK13* gene were decreased during skeletal muscle development.

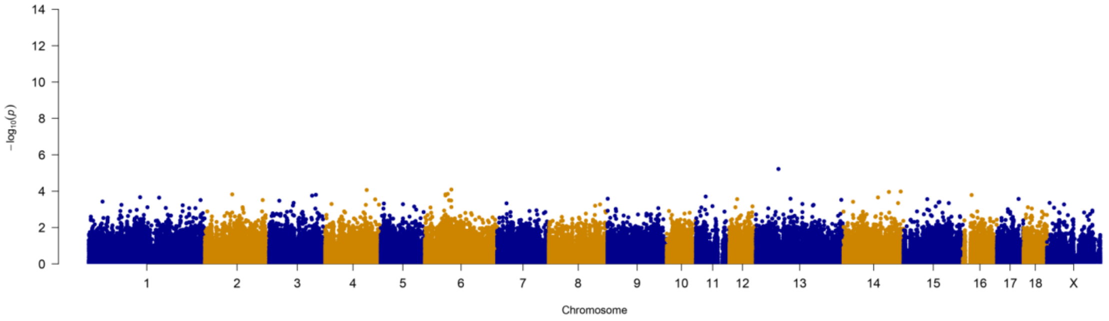

Supplementary Figure S11. Manhattan plot of genome wide P value of association between A-to-I editing and sex.

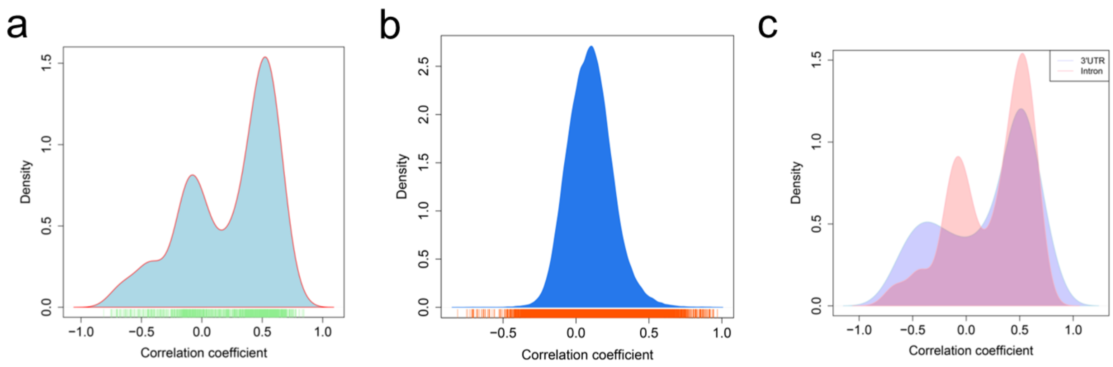

Supplementary Figure S12. Distribution of Pearson correlation coefficients of the editing rates with the expression level of its host mRNA. (**a**) All dDESs in genic regions. (**b**) All editing sites. (**c**) dDESs in 3’UTR and intron regions

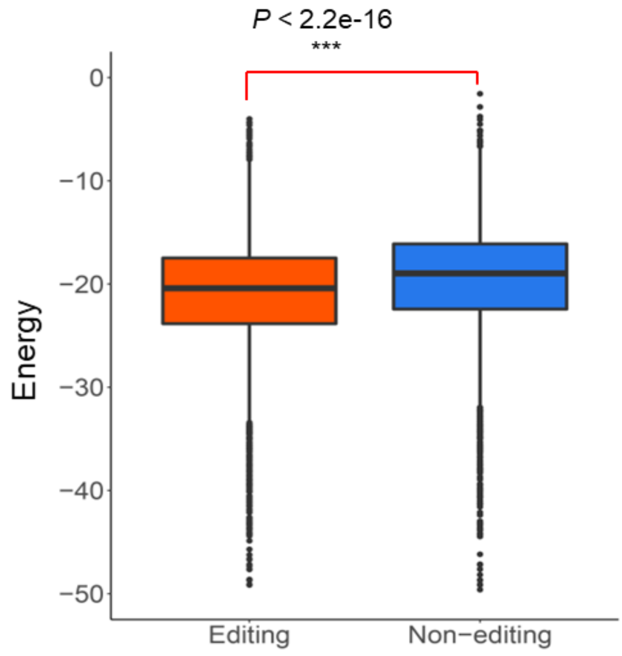

Supplementary Figure S13. Comparing the binding energy of miRNAs with flanking regions of sites with and without editing. The binding energies of miRNAs were predicted by miranda software with default values. The *P* value was from a two-tailed Wilcoxon rant sum test.

References

1. Ramaswami, G. & Li, J.B. RADAR: a rigorously annotated database of A-to-I RNA editing. *Nucleic Acids Research* **42**, D109-D113 (2014).
2. Picardi, E., D'Erchia, A.M., Lo Giudice, C. & Pesole, G. REDIportal: a comprehensive database of A-to-I RNA editing events in humans. *Nucleic Acids Research* **45**, D750-D757 (2017).
3. Tang, Z. *et al.* Comprehensive analysis of long non-coding RNAs highlights their spatio-temporal expression patterns and evolutional conservation in Sus scrofa. *Sci Rep* **7**, 43166 (2017).
4. Liang, G., Yang, Y., Niu, G., Tang, Z. & Li, K. Genome-wide profiling of Sus scrofa circular RNAs across nine organs and three developmental stages. *DNA Res* **24**, 523-535 (2017).
