# Supplementary material for: Developmental atlas of the RNA editome in Sus scrofa skeletal muscle
Source: DNA Res. 2019 Apr 23;26(3):261–72. doi: 10.1093/dnares/dsz006 (PMC6589548; doi:10.1093/dnares/dsz006)
Supplement: dsz006_Supplementary_Data [file dsz006_supplementary_data.zip › dsz006-Suppl_data/Supplementary Table S1.pdf]

**Supplementary Table S1.** Primer information for RNA editing validation

| Gene | Primer sequences (5'-3') |
|---|---|
| *ACTN2* | F: GCCTGCATGGCAGAAGAGAT<br>R: ACACCACAGGATCTGCCTTT |
| *AVPR1A* | F: GCTATCACCGTCCAGTTGACA<br>R: CACTGCTATGGCATGGGTTTG |
| *C2orf49* | F: CCTACAGAGCCGATGAGGGA<br>R: TTATCTGCAGGGCTGATGTCT |
| *EIF2AK2* | F: GCGCCTGAATAGTGGGAGAG<br>R: CTTGAGCAGATCTGTGAGGCT |
| *ENSSSCG00000039004-1* | F: GCTGTGTTAAAATGGGCCTGT<br>R: TATGACCAGCGCCACAAACA |
| *ENSSSCG00000039004-2* | F: ATTAGAGGTCATTTAGAGCCACTGT<br>R: GCTGACTATTGCTGGTGTGTT |
| *EYA4* | F: CTCAAAGCCACCTGGCAAAC<br>R: CTGTCCACTAAGGCCGTGTT |
| *H6PD* | F: ACTTATGGCAGGGGCTGTTC<br>R: TGAGCCTTGAGTTCAGCACA |
| *LUM* | F: CCCTACTCCCACCCCAGTTA<br>R: AGGAGTACAGGTGGGCAAGA |
| *MGAT4A* | F: AGGTGGGTTACAGGTGTGC<br>R: CTCAGAGCTGGCTACTTGGAA |
| *MTERF3* | F: GGGCGTCGAGCAATAGATGA<br>R: AGCCGTGGCATATAGGTTCC |
| *MYL12A* | F: GAACCTGGAAGCCCTTCTTGA<br>R: AGCATTGCATGTAGACGACTTT |
| *PHLPP2* | F: CTGCTCTCCTGGTGGTGTTT<br>R: TCCGCTTACCATCCAAGACG |
| *SASS6* | F: ACTTTGGAACTCCTCTGGCA<br>R: AGGTAGAGCAAATAGCACACTCA |
| *TNNT3* | F: GACAGTCTGGGGACAGCAAA<br>R: GACTCAGGTTCCTGTGAGGC |
| *VAMP5* | F: GATCCAAACCCTCTCTGCGA<br>R: TTTACAGCCCCCAAACCGAC |
| *ZBTB25* | F: CGGGGTCATTCTCTGCACC<br>R: AGCAGTTCAGGGCATAGTCG |
| *ZBTB8OS* | F: AGTTCTCCCAGGCTGCTCTA<br>R: CCTCTTCCTTTTGAGAAGAAACCAT |
