# Supplementary material for: Developmental atlas of the RNA editome in Sus scrofa skeletal muscle
Source: DNA Res. 2019 Apr 23;26(3):261–72. doi: 10.1093/dnares/dsz006 (PMC6589548; doi:10.1093/dnares/dsz006)
Supplement: dsz006_Supplementary_Data [file dsz006_supplementary_data.zip › dsz006-Suppl_data/Supplementary Table S2.pdf]

Supplementary Table S2. Primer information of vector construction

Name	Primer sequences (5'-3')
ACTN2$^{14:54710988}$-WT	F: CGAGCTCCTACAGCTGCCGGCCTA
	R: CCGCTCGAGTAACACCACCTTGGCACTTG
ACTN2$^{14:54710988}$-ET	F: CATGGATGCTAGTCAAATTCATGTCCACTGAACCA
	R: AGGATGCAGGTTTGATCCCTGGCCTCACTCAGAGG
TMX4$^{17:16833004}$-WT	F: CGAGCTCCTACAGAACGCAACCCAAAT
	R: CCGCTCGAGCTTCCCTGTTACAATGTCAGTTAT
TMX4$^{17:16833004}$-ET	F: TATCTAATTTTGATATATATAAAATTCTATAATTA
	R: AATTAAATAAAACTAGACTTTGGAACAGGGGTATA
AVPR1A$^{5:27827411}$-WT	F: CGAGCTCGTTAAGGATCTGTTGTTGTCAC
	R: CCGCTCGAGGAACTTTCCACTTTTCACTCT
AVPR1A$^{5:27827411}$-ET	F: TTATGCCCCTACATGATAGGACTTCTAAACCAATC
	R: TTTTTAACACAGTGAGCCCATCATCAGGCTGAGGT
MIB1$^{6:107039734}$-WT	F: CGAGCTCCCGTATAGGAGTTCCCGTCAT
	R: CGAGCTCGCCACACCTGCGGCATAT
MIB1$^{6:107039734}$-ET	F: GCCTTACTCAGTGGGTTGAGGATCCGGCGTTGCTG
	R: AGGGATTGAACCTGCATCCGCATGGGTAACAGTCA

Digestion sites are underlined.

WT: Wild type

ET: Editing type
